# Theoretical analysis of high-efficient dielectric nanofocusing for the generation of a brightness light source

**DOI:** 10.1038/s41598-019-44691-5

**Published:** 2019-06-03

**Authors:** Changhoon Park, Seonghyeon Oh, Jae W. Hahn

**Affiliations:** Nano Photonics Laboratory, School of Mechanical Engineering, Yonsei University, 50 Yonsei-ro, Seodaemun-gu, Seoul 120-749 Republic of Korea

**Keywords:** Nanophotonics and plasmonics, Sub-wavelength optics

## Abstract

High-brightness light sources with nanoscale volume are required in nonlinear physics studies or various nanoscale engineering areas. Although several plasmonic devices, such as plasmonic nanofocusing, have been proposed for light concentration, the efficient enhancement of the nanofocusing device to get a bright light source is still limited owing to the inevitable Ohmic loss resulting from high field confinement on metallic surface. We propose the concept of dielectric nanofocusing by reversing the concept of conventional plasmonic nanofocusing and using a three-dimensional bowtie nanoaperture (3D BNA). The optical simulations demonstrate that the 3D BNA can achieve an intensity enhancement factor of 9.01 × 10^4^. We calculate the dispersion relation for a tapered silver–SiN_x_–air waveguide to prove the possibility of focusing even for a high tapered angle. The theoretically calculated modal length can explain the origin of the high intensity enhancement by proving an energy flow from the dielectric layer to the air regime in dielectric nanofocusing. The performed optical and thermal simulations demonstrate that the 3D BNA can achieve a peak intensity of 6.21 PW/cm^2^ by avoiding the energy confinement around the metal. Our approach provides a new method for obtaining a high brightness light source.

## Introduction

A high-brightness light source is required in technologies based on nonlinear physics, such as nonlinear scattering^1^ and high-harmonic generation (HHG)^2^. Conventional approaches devised to obtain a high-brightness light source use a chirp pulse amplifier with a femtosecond laser^3,4^. Concurrently, for obtaining a high-brightness light source on a compact scale for further applications such as single molecule detection^5^, optical lithography^6,7^, and optical tweezers^8^, plasmonic devices exploiting surface plasmon polaritons (SPPs), mixed mode of an electromagnetic (EM) wave and heavy-ion phonon waves, have been proposed. This is owing to their high confinement factor and it demonstrated feasibility of optical hot spot generation^9,10^ or an HHG source^11,12^.

Plasmonic nanofocusing, which focuses SPPs onto the tip apex in a tapered structure, has been utilized for light concentration owing to the high energy confinement at the tip^13,14^. In plasmonic nanofocusing, because the group velocity of the SPPs converge to zero at the vicinity of the tip, the energy density of the SPPs diverges and their optical behaviour leads to a high intensity enhancement within a nanoscale volume^15^. Considering that the propagation of the EM mode in a tapered structure continuously changes the wavenumber or effective index, plasmonic nanofocusing may include some part of the reflection or scattering loss^15,16^. Because the effective index of an SPP diverges at the tip in a tapered structure, a plasmonic nanofocusing device requires a small tapered angle to suppress the reflection loss. It also requires a sharp structure to obtain better convergence of the group velocity to zero for achieving a high intensity enhancement^17^.

Because a conventional plasmonic device, including plasmonic nanofocusing, confines the optical energy on the metallic surface, a large portion of it is inevitably converted to Ohmic loss. This results in a degradation of the transmission efficiency or a high temperature increase under high-power laser illumination^18,19^. In addition, the optical performance of nanofocusing significantly depends on a sharp extremity of a tapered waveguide^17^. Considering the harsh requirements for achieving a sharp extremity and small tapered angle, plasmonic nanofocusing is fundamentally limited by the fabrication quality. In view of the energy losses, an appropriate device exhibiting a small Ohmic loss has yet to be proposed.

Here, we suggest the concept of dielectric nanofocusing for achieving a brightness light source by reversing the conventional concept of plasmonic nanofocusing, wherein the wavenumber of a waveguide or the group velocity converges to that of the air in a tapered metal/insulator/air/insulator/metal (MIAIM) structure. Owing to the convergence of the wavenumber in the vicinity of the aperture, dielectric nanofocusing allows even a high tapered angle to hold the adiabatic condition and is relatively insensitive to the sharpness of the structure. Unlike plasmon nanofocusing, in which there is a quite small variation in the modal length, dielectric nanofocusing controls the modal length of the EM wave for intensity enhancement. Specifically, as the optical energy inside the dielectric layer is transferred to the air channel at the tip of the aperture in a tapered MIAIM structure, dielectric nanofocusing leads to a high intensity enhancement as well as suppression of the intensity at the metallic surface, inducing a small Ohmic loss or heating effect. To demonstrate the feasibility of dielectric nanofocusing, we design a three-dimensional (3D) bowtie nano-aperture (BNA) to achieve extraordinarily high cut-off frequency and intensity enhancement. For the validation of dielectric nanofocusing using the 3D BNA, we theoretically calculate the modal length in the MIAIM structure, which is strongly related to the field enhancement, and show that the modal length converges at the aperture. To verify the high field enhancement and extraordinarily high cut-off frequency, we employ the finite-difference time-domain (FDTD) method. Furthermore, to prove the low loss of the 3D BNA, which are indispensable requirements for a brightness light source, we calculate the temperature distribution using the finite element method (FEM) and evaluate the performance of the 3D BNA compared to a two-dimensional (2D) BNA and nanofocusing.

## Results

### Device concept

**Figure 1:**
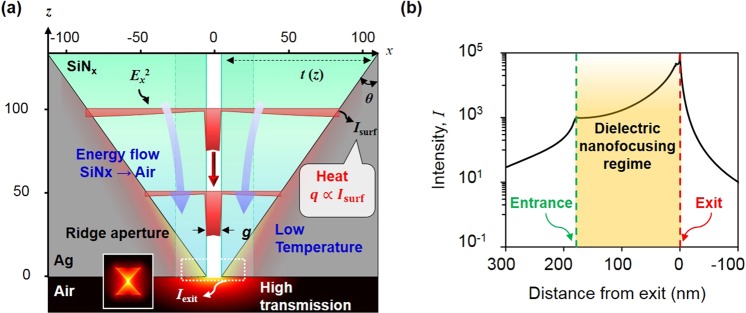
Concept of dielectric nanofocusing and intensity distribution around 3D BNA. (**a**) Schematic of 3D BNA composed of a tapered MIAIM waveguide whose tip apex is perforated in the shape of a bowtie hole. Red solid curve shows the theoretical intensity distribution of the TM_0_ mode of two un-coupled MIA waveguides at *z* = 100 nm and 50 nm, which is normalized by the maximum intensity at each z. (**b**) Intensity enhancement along the *z*-axis in real scale(blue solid line) and log scale(black solid line). Green and red dotted line implies location of MIAIM waveguide entrance and exit aperture respectively and dielectric nanofocusing occurs in yellow shaded region.

The 3D BNA is composed of a thin silicon nitride film deposited on a metallic tapered structure, whose tip apex is perforated with a bowtie shape, as illustrated in Fig. 1(a). To maximize the field enhancement factor with suppression of heat, the device consists of two parts: (i) a tapered MIAIM waveguide for dielectric nanofocusing and (ii) a BNA for extraordinarily high cut-off frequency and antenna resonance at the exit aperture.

At the entrance, the bulk illumination is coupled to the tapered MIAIM waveguide at the dielectric edge. For a MIAIM waveguide, high intensity is confined in the air channel owing to the large electric field discontinuity at the interface of the air/SiN_x_ layer^20^. As the guiding wave propagates toward the exit aperture, there is a decrease in the SiN_x_ lateral thickness, *t*(*z*), defined as *b*(*z*)-*a*, where *b*(*z*) is the location of the SiN_x_/metal boundary in the *x*-direction, and *a* is half of the gap size, *g*, leading to a variation in wavevector *β* or modal length *L*, a measure of the one-dimensional (1D) spot size. The tapered structure of the 3D BNA can act as a concentrator without experiencing reflection or scattering if the variations in the effective index of the tapered MIAIM waveguide, defined as *n*_eff_ = (*β*/*k*_0_), are sufficiently small to be within the distance of one wavelength, similar to the anti-reflection structure based on gradient refractive index optics^16^. To achieve this condition quite easily, we reverse the principle of conventional plasmonic nanofocusing by converging the effective index of the waveguide to that of the air at the exit aperture, which circumvents the rapid variation in the effective index.

In addition, the direction of the power flow converges to that of the air channel in the vicinity of the exit aperture. This implies that the optical energy stored in the SiN_x_ layer is gradually delivered to the air channel, which results in the compression of the modal length and concentration of optical energy at the exit. We define this phenomenon as dielectric nanofocusing. At the exit aperture, the sharp tapered structure induces a lightning rod effect around the tip and an antenna resonance due to the bowtie shape, maximizing the field concentration at the resonant frequency.

Concurrently, the intensity inside the metal is quite low compared to that in other regions owing to the presence of another electric field discontinuity at the metal/SiN_x_ boundary. This low intensity in the metal region prevents heat accumulation because the heat source is directly proportional to the intensity in the metal. Consequently, the peak temperature in the 3D BNA under laser illumination is relatively low compared to that in other purely metal-based plasmonic structures, and there is achievement of a high intensity enhancement simultaneously.

For validating the possibility of high-intensity enhancement factor in 3D BNA, we assume an axial thickness *T* of 170 nm, bowtie outline of 150 nm, and gap size of 10 nm, which leads to a resonant wavelength of 926 nm. Note that the resonant wavelength can be tuned by changing the bowtie outline, with an axial thickness T for which there is no theoretical cutoff (detailed explanation about the cutoff in waveguide is provided in the next section). Figure 1(b) shows the intensity distribution around 3D BNA, visualizing the energy concentration at the exit aperture. As seen in Fig. 1(b), coupling at the MIAIM waveguide entrance leads to an intensity enhancement owing to the E-field disparity between SiN_x_ and air. At the exit aperture, the intensity enhancement reaches 9.01 × 10^4^, which is 4–5 times higher than the intensity enhancement of nanofocusing with a gap or a tip radius of 2 nm^14,15^. Bowtie shaped hole at the exit acts as an antenna and its spectral performance is affected by bowtie outline. At the resonance of 926 nm with outline of 150 nm, 7% variation of a bowtie outline yields 80% of a maximum performance. In addition, at the resonance of 926 nm, FWHM in spectral range is approximately 73 nm

After passing through the exit aperture, the decay rate of the intensity in the z-direction is mainly determined by the quasi-spherical wave and evanescent wave along the metallic surface^21^. For a long distance from the exit aperture, the contribution of the evanescent wave to the transmission converges to zero and the decay rate of intensity is dominated by the quasi-spherical wave.

### Concept of dielectric nanofocusing and optical analysis of 3D BNA

To solve the dispersion relation in the MIAIM geometry, we consider the same boundary condition for solving a three-layer slab waveguide^22^. To investigate whether there is coupling between two symmetric MIA waveguide, the dispersion relations for two coupled waveguides, denoted by the MIAIM TM mode, and two un-coupled MIA waveguides, defined as the MIA TM mode, are calculated respectively. The dispersion relation of the un-coupled MIA waveguides in the TM mode can be expressed as (Supplementary Material, Section 1)

1 $$\tan ({k}_{{\rm{Si}}}(b-a)+\varphi -n\pi )=-\,\frac{{k}_{{\rm{Si}}}{\varepsilon }_{{\rm{Ag}}}}{{\varepsilon }_{{\rm{Si}}}{\gamma }_{{\rm{Ag}}}}$$

where *a* is a half of the gap size, *b*(*z*) is the metal/insulator boundary at certain *z* which is the same as *t*(*z*) wh*a*, *ε* is permittivity, *n* is an integer representing the waveguide mode, *k* and *γ* are the spatial frequency and decay constant of the waveguide mode in the *z*-direction respectively, $\tan \,\varphi =\frac{{\varepsilon }_{{\rm{air}}}{k}_{{\rm{Si}}}}{{\varepsilon }_{{\rm{Si}}}{\gamma }_{{\rm{air}}}}$, and the spatial frequency for each layer follows the relation: $\beta =\sqrt{{\varepsilon }_{{\rm{Si}}}{k}_{0}^{2}-{k}_{{\rm{Si}}}^{2}}=\sqrt{{\varepsilon }_{{\rm{air}}}{k}_{0}^{2}+{\gamma }_{{\rm{air}}}^{2}}=\sqrt{{\varepsilon }_{{\rm{Ag}}}{k}_{0}^{2}+{\gamma }_{{\rm{Ag}}}^{2}}$.

**Figure 2:**
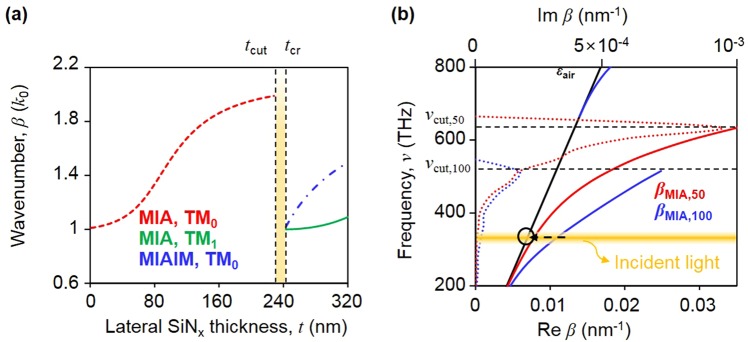
Dispersion relation in dielectric nanofocusing (**a**) wavenumber of a single MIA waveguide for the TM_0_ mode (red dotted line) and TM_1_ mode (green solid line) and MIAIM waveguide mode (blue dotted-solid line), where two symmetric MIA waveguide modes are coupled, with respect to the lateral SiN_x_ thickness at wavelength of 926 nm. (**b**) Dispersion relation of a single MIA waveguide for the TM mode with a lateral SiN_x_ thickness of 50 nm (red solid line for a real wavenumber and red dotted line for an imaginary wavenumber) and 100 nm (blue solid line for a real wavenumber and blue dotted line for an imaginary wavenumber) and MIAIM waveguide for the TM_0_ mode (green solid line). The black solid line shows the light cone in the air, and the horizontal black dotted line shows the cut-off frequency for the cases of *t* = 50 nm and 100 nm, respectively. Yellow line indicates the dispersion relation of the incident light, which is assumed to have a resonant wavelength of 926 nm.

**Figure 3:**
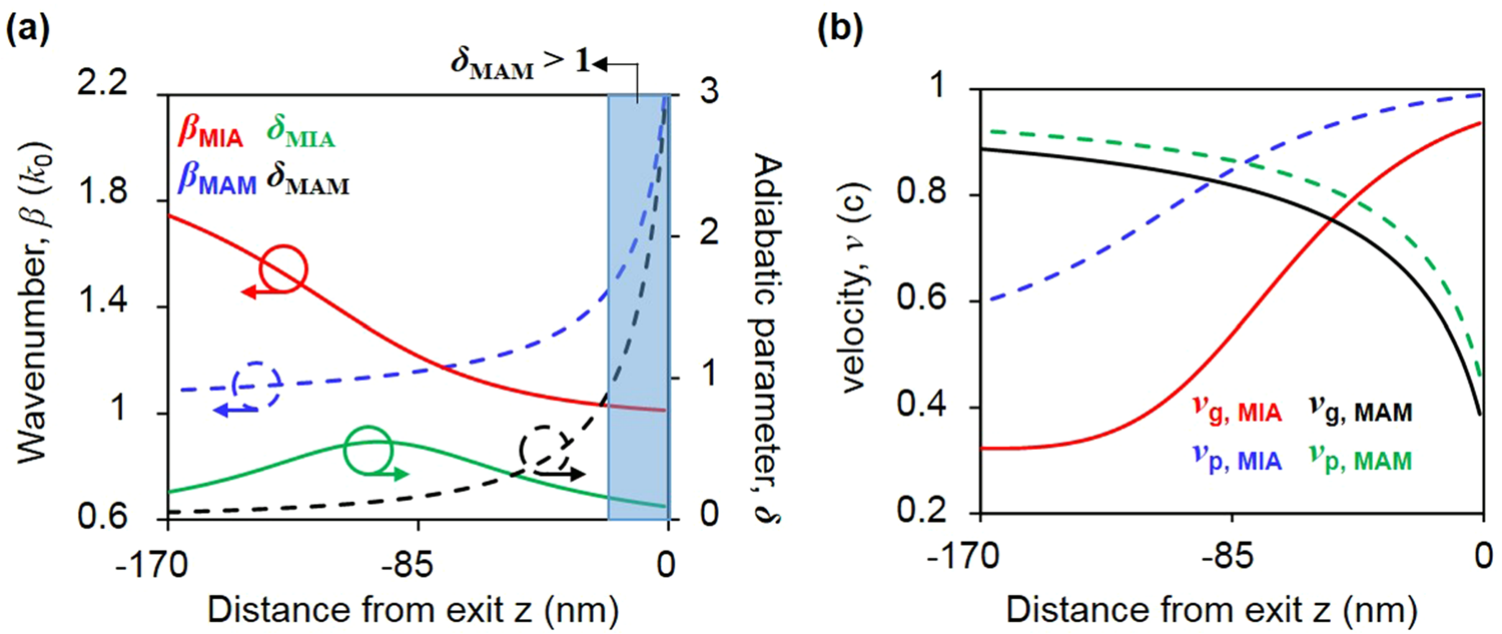
Comparison of the wavenumber, adiabatic parameter, phase velocity, and group velocity of dielectric nanofocusing (MIA waveguide) and plasmonic nanofocusing (MAM waveguide). (**a**) wavenumber (red solid line for the MIA waveguide and blue dotted line for the MAM waveguide) and adiabatic parameter (green solid line for the MIA waveguide and black dotted line for the MAM waveguide) for the MAM and MIA tapered waveguides in terms of *z*. (**b**) phase velocity, *v*_p_ (blue dotted line for the MIA waveguide and green dotted line for the MAM waveguide) and group velocity *v*_g_ (red solid line for the MIA waveguide and black solid line for the MAM waveguide) with respect to *z* in the MIA and MAM tapered waveguides. All the results are calculated with a tapered angle of 70.6°.

In order to calculate the dispersion relation in Figs 2 and 3, Eq. (1) is used for obtaining the wavenumber, adiabatic parameter, and velocity of the MIA TM mode. Note that lateral SiN_x_ varies with respect to *z*, implying a continuous change in the dispersion relation. In Fig. 2, we analyze the mode of the tapered MIAIM waveguides in terms of dielectric thickness.

Figure 2(a) shows the calculated wavenumber of the coupled MIA waveguide mode (MIAIM TM mode), and un-coupled MIA waveguides (MIA TM mode) in terms of *t*. As there is no solution for the MIAIM waveguide mode for *t*  A*t*_cr_, i.e., this waveguide mode cannot deliver energy to the exit aperture, the TM_0_ mode of MIAIM waveguide is not exploited for dielectric nanofocusing. In contrast, the fundamental mode of the un-coupled MIA waveguides exists for the condition where *t* < *t*_cut_, leading to the possibility of energy concentration. In a forbidden gap where *t*_cut_ < *t* < *t*_cr_, the imaginary value of β is dominant and delivery of the energy is significantly reduced. When wavelength is 926 nm, *ε*_SiNx_ is 3.97, *ε*_Ag_ is −46.78 × 3.768i^23^, and *a* is 5 nm, the calculated *t*_cut_ and *t*_cr_ are 228 nm and 243 nm, respectively.

Figure 2(b) displays the dispersion relation of the MIA TM mode and MIAIM TM mode at fixed *t*. At a fixed frequency below the cut-off frequency, even if the dispersion relation of the MIA waveguide has two solutions, namely, long-range SPPs with a positive group velocity and short-range SPPs with a negative group velocity, we only adopt the former in Fig. 2(b) neglecting short-range SPPs owing to the high dissipation rate^24^. In the MIA TM mode, because the imaginary value of the wavenumber is quite small compared to its real value except for in the vicinity of the cut-off frequency, the energy loss induced by the propagation can be suppressed in dielectric nanofocusing. Concurrently, a thick SiN*x* MIAIM waveguide corresponds to a low cut-off frequency of the MIA mode, which implies that the cut-off frequency in the tapered MIAIM waveguide is determined by the maximum SiN*x* thickness. Note that the value of Im*β* drastically increases near the cut-off frequency. Therefore, for efficient dielectric nanofocusing, the maximum lateral dielectric thickness needs to be restricted so that the operating frequency is far from the cut-off frequency, allowing the 3D BNA to concentrate light on the exit aperture while suppressing the energy loss.

Given that there is no coupling in the MIAIM waveguide with thin insulator layer, we only compare the MIA waveguide with the MAM waveguide as a representative case of plasmonic nanofocusing in Fig. 3. To concentrate the optical energy with a small loss, the reflection loss should be minimized. The degree to which the reflection loss occurs is determined by adiabatic parameter *δ*, defined as (d*β*^−1^/d*z*)^15^. Plasmonic nanofocusing cannot hold the adiabatic condition at a high tapered angle, e.g., 70.6° which is determined in the fabrication process according to the crystalline structure of silicon. However, owing to the divergence of the wavenumber, dielectric nanofocusing in the MIAIM tapered waveguide can hold the adiabatic parameter far below 1 for the overall region because of the convergence of the wavenumber at the exit aperture in Fig. 3(a). In dielectric nanofocusing, the adiabatic parameter for the Ag/SiN_x_/air/SiN_x_/Ag geometry with a tapered angle of 70.6° has a maximum value of 0.55 at *z* = 90 nm.

For a tapered structure satisfying the adiabatic condition, the amplitude of the wave can be obtained from the energy flux conservation, conservation of product of summation of all optical energy in xy-plane and group velocity of the wave at *z*:

2 $$f(z)={v}_{g}(z){\int }_{-\infty }^{\infty }W(x,y,z){\rm{d}}x{\rm{d}}y={\rm{const}}$$

where *v*_g_ = *∂w*/*∂k* is the local group velocity and *W* is the EM energy. In plasmonic nanofocusing, the group velocity in the vicinity of the aperture or tip apex converges to 0 and the total EM energy over the *xy*-plane diverges, maximizing the intensity enhancement. However, convergence of the group velocity to zero in the metal-air-metal(MAM) or insulator-metal-insulator(IMI) geometry strongly depends on tip radius or gap size^14,15^. This implies that a sharp extremity of the geometry should be defined in the fabrication process for achieving a high intensity enhancement. The group velocity in the silver/air/silver geometry is 0.38, which is not quite low value for a gap size of 10 nm, as shown in Fig. 3(b).

Contrary to plasmonic nanofocusing, the group velocity and phase velocity of dielectric nanofocusing converge to *k*_0_ when approaching the exit, leading to degradation of the intensity enhancement factor. However, the origin of the high intensity enhancement factor in dielectric nanofocusing is not the control of the group velocity but that of the modal length, *L*. Specifically, there is little variation in the modal length in plasmonic nanofocusing because the optical energy is practically confined in the vicinity of the metallic surface. However, the EM energy is distributed in the air channel, SiN_x_ layer, and metal region, which causes variation in the modal length with respect to *z*. Thus, even if there is degradation of the intensity enhancement owing to the increase in the group velocity in dielectric nanofocusing, there is a decrease in the integral range, except for the metallic region in Eq. (2), which physically implies *L*, leading to a high energy density of the optical spot.

To explain the intensity enhancement factor in dielectric nanofocusing, we adopt the mathematical form of the modal length, defined as ${\int }_{-\infty }^{\infty }\frac{d(\varepsilon (x)w)}{dw}{|E(x)|}^{2}\,/{\rm{\max }}\{\frac{d(\varepsilon (x)w)}{dw}{|E(x)|}^{2}\}$| with neglect of the magnetic energy. Given that product of the group velocity and the total optical energy over the *xy*-plane is conserved at each *z*, decreasing *L* increases the intensity enhancement factor. For the condition where the ridge gap size is much smaller than the wavelength, the analytical expression of *L* for the MIAIM waveguide can be expressed as (Supplementary Material, Section 2)

3 $$L=2a+\frac{{\varepsilon }_{{\rm{air}}}}{{\varepsilon }_{{\rm{Si}}}{\sin }^{2}\varphi }\{(b-a)-\frac{1}{2{k}_{{\rm{Si}}}}[\sin (2{k}_{{\rm{Si}}}(b-a)+2\varphi )-\,\sin \,2\varphi ]\}$$

When approaching the exit aperture, *L*(*z*) decreases and converges to 2*a* because the term in the bracket in Eq. (3) converges to 0, compressing the optical energy and maximizing the intensity enhancement. In addition, while the modal length originating from the air channel remains practically unchanged for all *z*, the modal length resulting from SiN_x_ layer, which is the second term in Eq. (3), continuously varies with respect to *z*. This implies that the optical energy inside SiN_x_ is the main source of the high intensity enhancement in dielectric nanofocusing.

**Figure 4:**
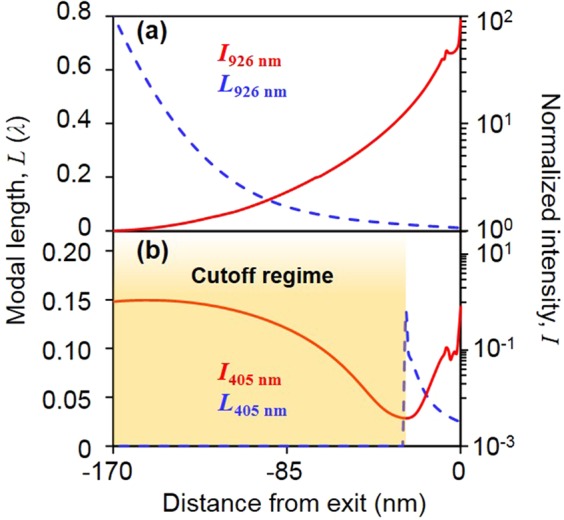
Calculated modal length(blue dotted line) and intensity enhancement(red solid line) (**a**) at resonant wavelength of 926 nm and (**b**) wavelength of 405 nm. All the calculated intensity enhancement is normalized by intensity at the entrance of MIAIM waveguide respectively (green dotted line in Fig. 1).

In Fig. 4(a), at the resonant wavelength of the 3D BNA, the cut-off frequency for the overall region is higher than the operating frequency. This occurs when the adiabatic condition being held automatically because the adiabatic parameter is below 1 and *I*(*z*) is continuously increasing when approaching the exit aperture, owing to compression of the modal length. Contrary to the resonant case where the wavelength is below the resonant wavelength, in this case, *λ* = 405 nm, and so, the reflection or scattering loss is predominant for a thick *t* as a thicker SiN_x_ layer leads to a lower cut-off frequency. After a certain point, *z* = −27 nm in this case, the intensity enhancement increases when the operating frequency is smaller than the cut-off frequency of the MIA TM mode. Given that the cutoff point predicted by the FDTD simulation is *z* = −31 nm (local minimum point on the red solid-line), which is as close as that predicted by the dispersion relation (local maximum point on the blue dotted line), the dielectric nanofocusing MIAIM waveguide with thin film structure is well described by the MIA TM mode dispersion.

In Fig. 4, there is small fluctuation of intensity enhancement in the vicinity of exit aperture. Note that the perforation of bowtie shape hole onto tapered MIAIM structure makes unintentional MAM structure around the exit and the length of MAM structure is given by *a* × cot (*θ*). As a results, the transition from MIAIM mode to MAM mode might lead to radiation or scattering loss, giving rise to small kink in the intensity enhancement at *z* = −7 nm. Small gap size or large tapered angle can suppress this phenomenon.

### Thermal analysis of 3D BNA

To evaluate the optical loss and its effect, we perform FEM simulations for the thermal analysis. For the thermal analysis, we extract the absorbed power from the FDTD simulation. Subsequently, the absorbed power is input into the thermal source of the FEM simulation, and the heat equation is solved by using the DEVICE program. Details related to thermal boundary condition and FDTD data of 3D BNA and 2D BNA for thermal analysis are described in Supplementary Material, Section 3.

To demonstrate the thermal performance of the 3D BNA, we adopt a 2D BNA as a reference and equate the brightness of the 3D BNA and 2D BNA at the exit by imposing different input laser powers. Specifically, because the intensity enhancement factor of the 3D BNA is 9 times higher than that of the 2D BNA at a gap size of 10 nm, the incident laser intensity on the 3D BNA is 2.55 × 10^−2^ TW/(cm)^2^, which is 0.11 times lower than the used power for 2D BNA. As illustrated in Fig. 3a, the local temperature for both the 3D BNA and 2D BNA reaches the peak at the exit owing to the high intensity enhancement in the metal, resulting in a maximum value of *q*.

**Figure 5:**
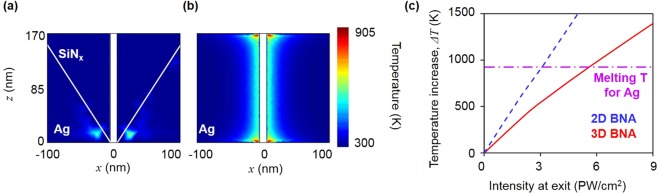
Thermal characteristics of the 3D BNA (**a**) Temperature distribution around the 3D BNA and (**b**) 2D BNA under the same condition where the intensity at the exit is 2.3 PW/(cm)^2^ (**c**) Temperature increases with respect to the intensity at the exit in the 3D BNA (blue dotted line) and 2D BNA (red solid line).

Even if the 2D BNA and 3D BNA have the same brightness factor of 2.3 PW/(cm)^2^ in Fig. 5(a), the calculated maximum temperature of the 2D BNA is expected to be 1.45 times higher than that of the 3D BNA. Contrary to the 2D BNA, whose maximum intensity distribution exists at the metallic surface, which leads to a high Ohmic loss or heat source, the optical energy in 3D BNA is highly concentrated around the air gap region, suppressing the Ohmic loss. Considering that a conventional plasmonic device, including plasmonic nanofocusing, shows maximum intensity around the metallic surface and in turn, induces a large Ohmic loss, dielectric nanofocusing is suitable for providing a brightness source contrary to other plasmonic devices. Based on the FEM simulation results, the maximum acceptable intensity not exceeding the melting temperature of silver for 3D BNA is 6.78 PW/(cm)^2^ which is 1.88 times higher than that of the 2D BNA, as shown in Fig. 5(b). Although additional damage originating from pulse laser such as non-thermal photoemission of ions and electrons can induce surface cracking^25^, which implies that the maximum acceptable intensity calculated in this study may not be accurate, our theoretical approach still can explain superiority of thermal durability.

## Discussion

In this study, we have proposed a concept of dielectric nanofocusing by employing a tapered MIAIM structure. Unlike conventional dielectric nanofocusing, the proposed technique exploits the convergence of the wavenumber at the exit aperture, leading to sufficient adiabatic condition. Using this method, in this study, the maximum value of the adiabatic parameter is 0.55 even for a large tapered angle of 70.6°. For efficient dielectric nanofocusing, we have proposed a 3D BNA, which achieves an enhancement factor of 9.01 × 10^4^ with a ridge gap size of 10 nm and axial SiN_x_ thickness, *T* of 170 nm. All the dimensions of 3D BNA in this study can be fabricated through the methodology published in our previous paper^26^ and a ridge gap size of 10 nm can be achievable by using helium ion microscopy.

To demonstrate the small Ohmic loss in the 3D BNA, we analyse the thermal characteristics of the 3D BNA by performing the FDTD and FEM simulation simultaneously. We show that the maximum achievable intensity at the exit aperture of 3D BNA is 6.21 PW/(cm)^2^. We demonstrate that the 3D BNA exhibits the highest intensity enhancement and a high thermal threshold compared to other plasmonic devices within the achievable gap size.

We expect that the concept of dielectric nanofocusing can be practically applied to light concentration devices owing to the small adiabatic parameter in a large tapered angle. Moreover, we believe that the 3D BNA with a high field confinement can be applied to imaging single molecules, optical tweezers, and magnetic recording, by minimizing the sample damage or heat expansion of the aperture. Specifically, considering possible thermal damage by femtosecond laser in HHG experiment and efficiency of HHG strongly depends on the light intensity, 3D BNA may be adequate for HHG application in respect of thermal and optical point of view. In addition, its high intensity enhancement consisting of relatively high radiative enhancement and low non-radiative enhancement, which is expected by suppression of Ohmic loss, may enable high spontaneous emission rate enhancement or Purcell factor with a single emitter. Furthermore, we expect that the configuration of the 3D BNA will be helpful in providing a visual design for the design of a non-thermal plasmonic system.

## Supplementary information

Supplementary information
