# Supplementary material for: Theoretical analysis of high-efficient dielectric nanofocusing for the generation of a brightness light source
Source: Sci Rep. 2019 Jun 3;9:8207. doi: 10.1038/s41598-019-44691-5 (PMC6547690; doi:10.1038/s41598-019-44691-5)
Supplement: Supplementary file 1 — Supplementary information [file 41598_2019_44691_MOESM1_ESM.docx]

**Supplementary information**

**Theoretical analysis of high-efficient dielectric nanofocusing for the generation of a brightness light source**

Changhoon Park, Seonghyeon Oh, and Jae W. Hahn*

Nano Photonics Laboratory, School of Mechanical Engineering, Yonsei University,

50 Yonsei-ro, Seodaemun-gu, Seoul 120-749, Republic of Korea

*E-mail: [jaewhahn@yonsei.ac.kr](mailto:jaewhahn@yonsei.ac.kr)

**1. Dispersion relation in MIAIM structure**

In the tapered metal-insulator-air-insulatr-metal(MIAIM) structure, the intensity distribution of the transverse magnetic (TM) mode along the *x* axis continuously varies with respect to the *z* coordinate. In this section, we analytically derive the intensity distribution by solving the Helmholtz equation with following boundary conditions: (1) the electromagnetic (EM) field is confined at the SiN_x_/air interface, resulting in near-field characteristics in the *x*-direction in the air regime; (2) the EM field in SiN_x_ has a real value of the wavevector in all directions, supporting the propagating mode; and (3) the EM field exponentially decreases in the *x*-direction owing to metal conductivity. These conditions are summarized by

$H_{y}=\left\{\begin{matrix} A_{1}\exp[\gamma_{\text{air}}(\left|x\right|-a)] \\ A_{2}\cos[k_{\text{SiNx}}(\left|x\right|-a)]+B_{2}[\sin k_{\text{SiNx}}(\left|x\right|-a)] \\ A_{3}\exp[-\gamma_{\text{Ag}}(\left|x\right|-b)] \end{matrix}\right. \begin{matrix} \text{ }\left|x\right|<a \\ a\leq\left|x\right|<b \\ \text{ }b\leq\left|x\right| \end{matrix}$ (S1)

where *A*_1_, *A*_2_, *B*_2_, *A*_3_ are amplitude constant of each region, *a* is half of gap size, *b* is the location of SiN_x_/silver interface, and the spatial frequency obeys the following relation: $\beta=\sqrt{{\varepsilon_{\mathrm{SiNx}}k}_{0}^{2}-k_{\mathrm{SiNx}}^{2}}=\sqrt{{\varepsilon_{\mathrm{air}}k}_{0}^{2}+\gamma_{\mathrm{air}}^{2}}=\sqrt{{\varepsilon_{\mathrm{ag}}k}_{0}^{2}+\gamma_{\mathrm{Ag}}^{2}}$.

**Figure S1.** Schematic of the MIAIM waveguide

By applying the *E*_z_ field and *H*_y_ field continuity at *x* = *a* and *x* = *b*, one can obtain

$H_{y}=A\left\{\begin{matrix} \exp[\gamma_{\text{air}}(\left|x\right|-a)] \left|x\right|<a \\ \cos[k_{\text{Si}}(\left|x\right|-a)]+\frac{\varepsilon_{\text{Si}}\gamma_{\text{air}}}{\varepsilon_{\text{air}}k_{\text{Si}}}\sin[k_{\text{Si}}(\left|x\right|-a)] a\leq\left|x\right|<b \\ \left\{\cos[k_{\text{Si}}(b-a)]+\frac{\varepsilon_{\text{SiNx}}\gamma_{\text{air}}}{\varepsilon_{\text{air}}k_{\text{SiNx}}}\sin[k_{\text{Si}}(b-a)]\right\} \times\exp[-\gamma_{\text{A}g}(\left|x\right|-b)] b\leq\left|x\right| \end{matrix}\right.$ (S2)

where *A* is obtained by the normalization of the optical power., which is equal to *A*_1_. Because the EM mode in the MIAIM structure consists of two symmetric metal-insulator-air(MIA) waveguides that are uncoupled when (*b*-*a*) is smaller than the cutoff thickness, the analytic solution of the MIAIM waveguide can be obtained by the superposition of the two MIA waveguide modes:

 (S3)

where $\tan\phi= \frac{\varepsilon_{\mathrm{Si}}\gamma_{\mathrm{air}}}{\varepsilon_{\mathrm{air}}\gamma_{\mathrm{Si}}}$. To derive Eq. (S3), we apply a trigonometric relation to Eq. (S2). The dispersion relation of two symmetric MIA waveguides can be obtained from the *E_z_* field continuity at *x* = *b*:

 (S4)

For a high-order TM mode of the MIA waveguide, Eq. (S4) can be generalized as

 (S5)

where *n* is the integer corresponding to the TM*_n_* mode. For instance, the fundamental mode is for n = 0, first mode is for *n* = 1, and second mode is for *n* = 2.

**2. Analytical expression for modal length**

The intensity enhancement factor in dielectric nanofocusing can be obtained by using the energy flux conservation. Specifically, if energy except for the reflection loss or Ohmic loss is conserved at each *z*, then the modal length, a measure of the one-dimensional spot size, can be exploited to derive the intensity enhancement. Modal length of the EM mode is defined as

$L(z)=\frac{\int W(x)dx}{W_{\max}}=\frac{\int\varepsilon\left|E(x)\right|^{2}+\mu_{0}\left|H(x)\right|^{2}dx}{\varepsilon\left|E(x_{\max})\right|^{2}+\mu_{0}\left|H(x_{\max})\right|^{2}}$ (S6)

where *W* is the EM energy and *x*_max_ is the location where W is maxima in a given *z.* By using Eq. (S3), the EM energy distribution along the *x*-axis can be expressed as

 (S7)

At any z, W typically has a maximum value at *x* = *a* because of the significant electric field disparity at the dielectric/air boundary. In addition, given that the W inside a metal is quite small compared to the optical energy in both the air channel and SiN_x_, integration over the metal region is neglected in this work. Starting with these conditions, the integration of the EM energy over the air region can be expressed as

$\begin{matrix} L_{\text{air}}=\frac{2\int_{0}^{a} \frac{1}{\varepsilon_{\text{air}}}\exp[2\gamma_{\text{air}}(\left|x\right|-a)]dx}{\frac{1}{\varepsilon_{\text{air}}}} \\ =\frac{\exp(\gamma_{\text{air}}a)-\exp(-\gamma_{\text{air}}a)}{\gamma_{\text{air}}\exp(\gamma_{\text{air}}a)} \\ =\frac{2\sinh(\gamma_{\text{air}}a)}{\gamma_{\text{air}}\exp(\gamma_{\text{air}}a)}\approx2a \end{matrix}$ (S8)

Using the fact that *γ*_air_*a* ≪ 1 in 3D BNA, sinh(*x*) is well approximated as *x* when –*a* < *x* < *a*, leading to L_air_ (*z*) = 2*a* for all z. Using trigonometric relations, the integration of the optical energy over the SiN_x_ region can be expressed as

 (S9)

By adding Eq. (S8) and Eq. (S9), Eq. (3) in the manuscript can be obtained.

**3. FDTD simulation data of 3D and 2D bowtie aperture for thermal analysis**

In the manuscript, the outline of the 3D bowtie aperture and SiN_x_ thickness are set to be 150 nm and 170 nm, respectively. Concurrently, for the comparison with 3D bowtie at the same resonance wavelength, 926 nm in this case, the outline of the bowtie aperture and thickness of the metal are set to be 130 nm and 170 nm, respectively.

As shown in Fig. S2, the 3D BNA shows an intensity enhancement of 9.01× 10^4^, approximately 9 times higher than the intensity enhancement of 2D BNA for the same gap size of 10 nm. As the loss power density, or heat source determined from the Ohmic loss, *q*=1/2ε_0_ *w*Im(*ε*)|*E*|^2^, which implies that the heat source is proportional to the wave frequency, the resonant wavelengths of both apertures are equated in this study.

For thermal analysis, we set a convective boundary condition in both SiNx/air boundary at the upper layer and Ag/air at the bottom layer for 3D BNA, and Ag/air boundary at the upper layer and bottom layer for 2D BNA. Except for the upper or bottom layer, the conductive boundary condition is used for solving the heat transfer equation for both 3D BNA and 2D BNA.


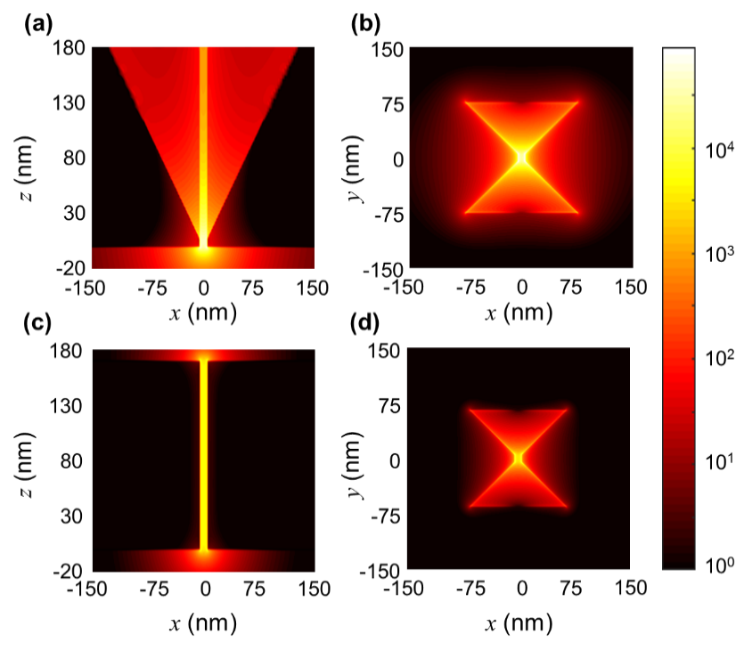


**Figure S3.** Intensity distribution of 3D BNA and 2D BNA at resonance wavelength. (a-b) Intensity distribution of 3D BNA in the xz plane(a) and xy plane(b). (c-d) Intensity distribution of 2D BNA in the xz plane(c) and xz plane(d). All the calculation results are plotted with log scale and normalized by the maximum intensity of the 3D BNA at the exit aperture, 9.01× 10^4^.

For solving the transient heat transfer equation, we set all the initial local temperatures of 3D BNA and 2D BNA as 300 K. For thermal analysis, we set a plane wave having a source size of 1.4 μm × 1.4 μm with an E-field of 1 V/m in the FDTD simulation. The calculated absorbed power with Ohmic loss is exploited for the heat source, and the input optical power of the incident beam is controlled by scaling the heat source in the thermal simulation.
